# Transcriptional Analysis of Lennert Lymphoma Reveals a Unique Profile and Identifies Novel Therapeutic Targets

**DOI:** 10.3389/fgene.2019.00780

**Published:** 2019-09-10

**Authors:** Maryam Etebari, Mohsen Navari, Claudio Agostinelli, Axel Visani, Cristiano Peron, Javeed Iqbal, Giorgio Inghirami, Pier Paolo Piccaluga

**Affiliations:** ^1^Department of Experimental, Diagnostic, and Specialty Medicine, Bologna University Medical School, Bologna, Italy; ^2^Department of Medical Biotechnology, School of Paramedical Sciences, Torbat Heydariyeh University of Medical Sciences, Torbat Heydariyeh, Iran; ^3^Research Center of Advanced Technologies in Medicine, Torbat Heydariyeh University of Medical Sciences, Torbat Heydariyeh, Iran; ^4^Bioinformatics Research Group, Mashhad University of Medical Sciences, Mashhad, Iran; ^5^Department of Pathology and Microbiology, University of Nebraska Medical Center, Omaha, NE, United States; ^6^Department of Pathology and Laboratory Medicine, Weill Cornell Medical College, New York, NY, United States; ^7^Department of Biomolecular Strategies, Genetics, Avant-Garde Therapies and Neurosciences (SBGN), Euro-Mediterranean Institute of Science and Technology (IEMEST), Palermo, Italy; ^8^School of Health, Department of Pathology, Jomo Kenyatta University of Agriculture and Technology, Nairobi, Kenya

**Keywords:** Lennert lymphoma, peripheral T-cell lymphoma—not otherwise specified, WHO classification, gene expression profiling, miRNA profiling

## Abstract

Lennert lymphoma (LL) is a lymphoepithelioid morphological variant of peripheral T-cell lymphoma—not otherwise specified (PTCL/NOS), clinically characterized by better prognosis if compared with other PTCL/NOS. Although well characterized as far as morphology and phenotype are concerned, very little is known regarding its molecular features. In this study, we investigated the transcriptional profile of this tumor aiming 1) to identify its cellular counterparts; 2) to better define its relation with other PTCLs—and, therefore, its possible position in lymphoma classification; and 3) to define pathogenetic mechanisms, possibly unveiling novel therapeutic targets. To address these issues, we performed gene and microRNA expression profiling on LL and other PTCL/NOS cases; we identified different genes and microRNAs that discriminated LL from other PTCL/NOS. Particularly, LL revealed a molecular signature significantly enriched in helper function and clearly distinguishable from other PTCL/NOS. Furthermore, PI3K/Akt/mTOR pathway emerged as novel potential therapeutic target. In conclusion, based on the already known particular morphological and clinical features, the new molecular findings support the hypothesis that LL might be classified as a separate entity. Preclinical and clinical studies testing the efficacy of PI3K/MTOR inhibitors in this setting are warranted.

## Introduction

Peripheral T-cell lymphomas (PTCLs) are a heterogeneous group of non-Hodgkin lymphomas. Although more than 25 different entities are currently listed in the World Health Organization (WHO) classification (Swerdlow et al., 2016; Swerdlow et al., 2017), the most common nodal PTCL subtypes include anaplastic large-cell lymphoma ALK+ and ALK-, angioimmunoblastic T-cell lymphoma and other T-follicular helper-derived tumors, and peripheral T-cell lymphoma—not otherwise specified (PTCL/NOS). The latter accounts for more than one third of the PTCLs, and it cannot be further classified due to lack of uniform characteristic criteria, thus making it a challenge in terms of both diagnosis and treatment choice (Zettl et al., 2004).

PTCL/NOS encompasses some morphological variants, including Lennert lymphoma (LL) and T-zone lymphomas (Dorfman et al., 2003; Swerdlow et al., 2017). LL was first described by Karl Lennert in 1952 and, later in 1968, by Lennert and Mestdagh (Lennert and Mestdagh, 1968). At first, the disease was classified as “lymphoepithelioid cellular lymphoma,” later on being recognized as a T-cell lymphoma (Feller et al., 1986). Since its discovery, the term “LL” has been used to describe a wide range of malignant lymphomas, all of which are characterized by the high level of epithelioid cells, although those lymphomas might actually correspond to different categories. In the fourth edition of the WHO classification, LL was finally classified as a PTCL/NOS morphological variant characterized by clear cell cytology, cytotoxic phenotype, and abundant, when not prominent, epithelioid cells reaction (Swerdlow et al., 2017). So far, only few publications have dealt with the biological nature of LL, most probably because of the very low incidence level. By means of immunohistochemistry (IHC), recent studies have discovered a prevalent expression of cytotoxic T-cell markers, e.g., granzyme B, and T-cell intracellular antigen-1 in LL, though usually in absence of cluster of differentiation 8 (CD8), this making questionable the LL cellular derivation (Hartmann et al., 2011). Importantly, LL was found to present different morphological and phenotypical features when compared with the other epithelioid-rich subtypes of PTCL, thus suggesting it as a possible distinct entity. In addition, a large cooperative study indicated that LL is probably provided with a particular, more favorable clinical behavior when compared with other PTCL/NOS (Weisenburger et al., 2011). By contrast, no data are available concerning LL molecular profile, thus leaving the issue of LL’s actual nature unsolved.

Bearing this in mind, we investigated the transcriptional profile of LL in terms of both genes and microRNAs (miRNAs) and compared it with those of other PTCL/NOS, in order to 1) assess its cellular counterpart; 2) better define its relation with other PTCLs, and 3) define pathogenic mechanisms, possibly unveiling novel therapeutic targets.

## Materials and Methods

### Case Series

We studied 80 formalin-fixed paraffin-embedded (FFPE) tumor cases including 68 PTCL/NOS and 12 LL cases, collected at the Hematopathology Unit of S. Orsola Malpighi Hospital, Bologna University. All cases were studied by extensive IHC. The diagnosis was made by at least two expert hematopathologists according to the criteria of the WHO classification. Furthermore, we isolated CD4+ T-cells from healthy donors by means of MiniMACS system (Miltenyi Biotec, Bergisch Gladbach, Germany), as previously described (Piccaluga et al., 2007). The main clinico-pathological features are reported in Table 1.

**Table 1:** Patients’ characteristics. The significant p-values are indicated with bolded text.

	PTCL/NOS	Lennert Lymphoma	p-value
Number of cases	68	10*	
Male : Female	1:1	9:1	**0.02**
Mean age, years (range)	50.9 (7–80)	48.7 (22–67)	0.8
Stage III-IV (%)	47/68 (69%)	6/10 (60%)	0.7
B-symptoms (%)	33/68 (49%)	1/10 (10%)	**0.036**
Bulky disease (%)	7/68 (10%)	3/10 (30%)	0.11
Mediastinal mass (%)	21/68 (31%)	4/10 (40%)	0.7
Bone marrow involvement (%)	21/68 (31%)	1/10 (10%)	0.26
Extra-nodal involvement (%)	26/68 (38%)	2/10 (20%)	0.32
Hemophagocytic sindrome (%)	3/68 (4%)	0/10 (0%)	1
*Treatment response and survival*			
CR (%)	33/68 (49%)	6/10 (60%)	0.7
PR (%)	13/68 (19%)	3/10 (30%)	0.4
NR (%)	22/68 (32%)	1/10 (10%)	0.26
OR (%)	46/68 (67%)	9/10 (90%)	0.26
8-years OS (%)	29/68 (43%)	6/10 (60%)	0.3

*Clinical information was available for only 10 cases of Lennert Lymphoma.

The study was conducted according to the principles of the Helsinki Declaration after obtaining approval from the Internal Review Board (Prot. No. IRB_003_2011). Written informed consent was obtained from all patients for the tissue analysis.

### Gene and microRNA Expression Profile Generation

RecoverAll^™^ Total Nucleic Acid Isolation Kit (Thermofisher, Waltham, MA, USA) was used to extract total RNA from FFPE samples. Up to five 10-μm-thick sections were processed per reaction. The samples were rehydrated using a series of xylene and ethanol washes. Next, they were subjected to a rigorous protease digestion with an incubation time tailored for recovery of total RNA. RNA was purified using a rapid glass-fibre filter methodology that includes an on-filter DNase treatment and was eluted into the low-salt buffer provided. A NanoDrop spectrophotometer (Thermo Scientific, Wilmington, DE, USA) was used to quantify RNA content and assesses its purity in terms of 260/280 and 260/230 absorbance.

Extraction of RNA from normal CD4+ samples was achieved by means of TRIZOL (Thermofisher, Waltham, MA, USA) reagent as previously described (Navari et al., 2014) and was quantified and quality-controlled as those previously mentioned.

In order to generate gene expression profiles (GEPs), we included all the tumor and 10 normal CD4+ T-cell samples. The obtained RNA was then processed as for manufacturer’s instructions to produce biotinylated complementary DNA (cDNA). Thereafter, biotinylated cDNA was then annealed to the DASL Assay Pool (Illumina, San Diego, CA, USA) probe groups that contain oligonucleotides specifically designed to interrogate each target sequence in the transcripts. Following this, the correctly annealed, assay-specific, oligos were extended and ligated to generate amplifiable products. These templates were labeled during PCR amplification by including fluorescent primers in the reaction. The resulting PCR products were hybridized on the Illumina HumanHT-12 WG-DASL V4.0 R2 (Illumina, San Diego, CA, USA) expression beadchip and scanned using the iScan System (Illumina, San Diego, CA, USA) to determine the expression level of specific genes.

miRNA profiling generation was carried out on 23 cancer cases (LL, N = 3; PTCL/NOS, N = 20) and 12 normal CD4+ T-cells using the TaqMan Array Human MicroRNA Card A v2.0 (Life Technologies, Carlsbad, CA, USA). Briefly, 350 ng of total RNA for each sample was reverse-transcribed using the Megaplex RT stem-loop primers in a 7.5-μl reaction volume through the protocol’s default 40 cycle runs; 2.5 μl of each RT product was pre-amplified in a 25-μl reaction volume with Megaplex Pre-Amp primers to increase detection sensitivity according to manufacturer’s specifications. Pool A set was used in all steps, enabling specific cDNA synthesis of 377 human miRNAs (mirBase v.10.1) and 3 small RNA controls (RNA44, RNA48, MammU6). Nine microliters of fourfold diluted pre-amplified RT product was 100-fold diluted in the PCR reaction mix and amplified using TaqMan low-density arrays on a TaqMan Real-Time 7900HT (Life Technologies, Carlsbad, CA USA) according to the standard protocol.

### Bioinformatic Analysis

Gene and miRNA expression analysis was carried out using GeneSpring version GX 12 (Agilent, MI, Italy), as previously reported (Piccaluga et al., 2007; Piccaluga et al., 2008; Piccaluga et al., 2011; Piccaluga et al., 2013). In brief, in case of miRNA, data were first transformed based on ddCT method. Then, both data of miRNA and gene expression were imported into GeneSpring and normalized based on the following criteria—threshold: 1.0; logbase: 2; normalization: shift to 75 percentile; baseline transformation: median of all samples. The probes with missing values were excluded. Principal component analysis (PCA) was used to discriminate the different biological samples on the basis of the distances of a reduced set of new variables (principal components), using the top three principal components for depicting the results in three dimensions, as described (Piccaluga et al., 2007; Piccaluga et al., 2008; Piccaluga et al., 2011; Piccaluga et al., 2013). Unsupervised hierarchical clustering analysis (HCA) was generated using an algorithm based on Pearson correlation and the average-linkage method, as reported (Hartigan, 1975; Eisen et al., 1998). Differentially expressed genes and miRNAs between any two groups of samples were identified using the criteria including p-value and fold change. The resulting genes/miRNAs were used to produce supervised HCA as described previously.

Validated miRNA targets were obtained from miRTarBase version 7.0 (Chou et al., 2018). The CIBERSORT was adopted to evaluate the content of immune cells in the analyzed samples *in silico* (Newman et al., 2015). Pathway analysis was performed using Ingenuity^®^ Pathway Analysis (IPA, QIAGEN Inc., https://www.qiagenbioinformatics.com/products/ingenuitypathway-analysis) as previously reported (Perucca et al., 2017). Broad Institute’s Molecular Signatures Database online tool and Gene Set Enrichment Analysis software were used for investigating enrichment of gene signatures in terms of gene ontology biological processes, oncogenic signatures, immunologic signatures, and curated gene sets, filtering the results based on false discovery rate (FDR)-adjusted p-value (Subramanian et al., 2005; Liberzon et al., 2015).

Gene expression studies were conducted according to the minimum information about a microarray experiment guidelines. The data discussed in this publication have been deposited in the National Center for Biotechnology Information’s Gene Expression Omnibus (accession numbers: GSE132550 and GSE132736).

### Tissue Microarray Construction and Immunohistochemical Analysis

For tissue microarray construction, a slide stained with hematoxylin and eosin was prepared from each available paraffin block, and representative tumor regions were morphologically identified and marked on each slide. Tissue cylinders with a diameter of 1.0 mm were punched from the marked areas of each block and brought into a recipient paraffin block using a precision instrument, as previously described (Went et al., 2006). Four-micrometer-thick sections were cut from each recipient block and used for Giemsa or immunohistochemical stains. During the diagnostic revision, specific antibodies, representative of the diverse T-lymphocyte commitments, were studied, which included T-helper (CD4), T-cytotoxic (CD8, TIA1, PERF, and granzyme B), T-follicular helper (CD10, BCL6, CXCL13, and PD1), and T-reg (FOXP3), as described (Hartmann et al., 2011). Samples of LL (N = 12) were tested on full section for PDCD4 (Atlas, rabbit polyclonal), STAT5 (Santa Cruz, rabbit polyclonal), and CD68 (PGM-1) to validate and interpreter GEP data. Details of the antibodies used, antigen retrieval, and detection methods strategies were previously reported (Went et al., 2006; Hartmann et al., 2011).

Each section was evaluated by two experienced pathologists. Each observer estimated the number of positive cells.

## Results

### CIBERSORT Analysis Unveiled Possible Correspondence of LL to Helper T-Lymphocytes and M_0_–M_1_ Macrophages Involvement

**Figure 1:**
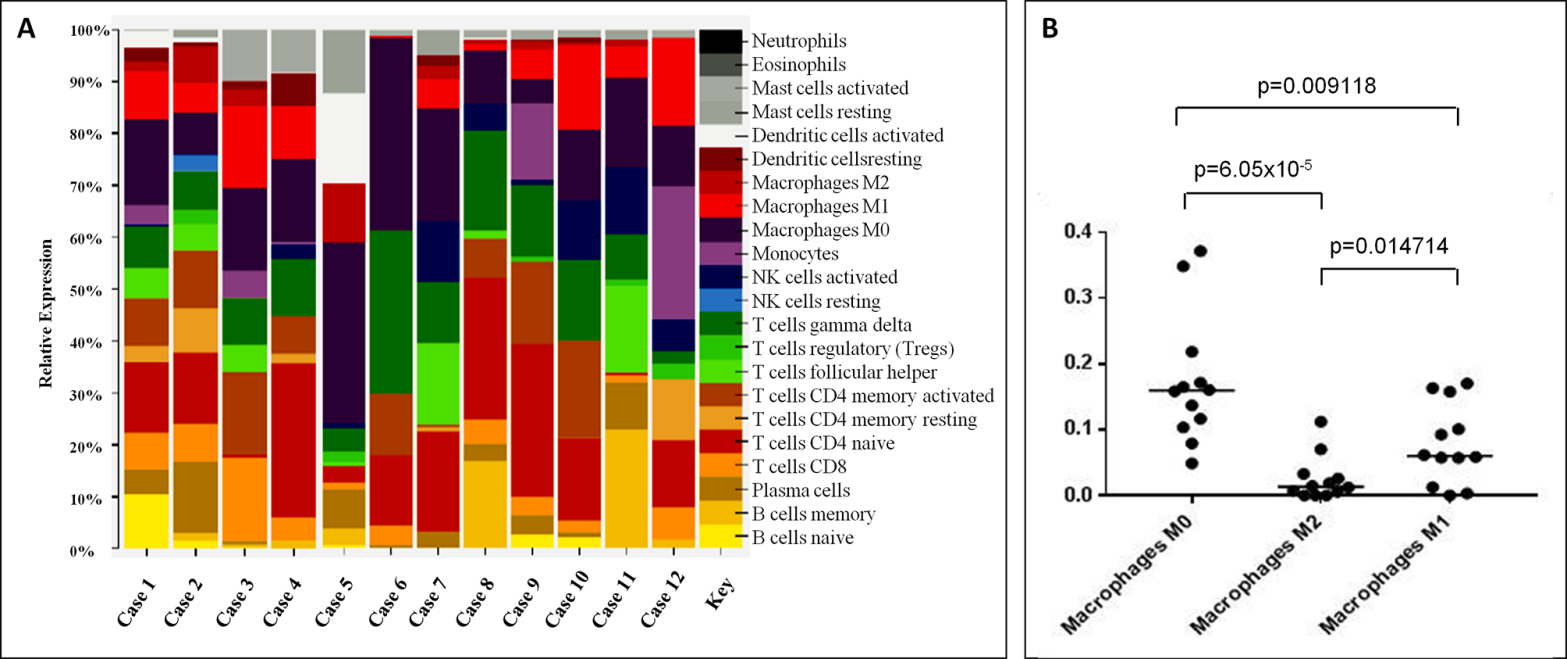
CIBERSORT analysis unveiled possible correspondence of Lennert lymphoma to helper T-lymphocytes and M_0_–M_1_ macrophages involvement. **(A)** CD4+ was the most represented cells; particularly, CD4 naïve profile was the most prevalent in 9/12 samples, CD4 memory activated profile was observed in 2/12 (in one case, being the CD8 profile equally abundant), and a T-follicular helper (T_FH_) profile in one. The proportions indicate the percentage for each cell population in the samples. **(B)** At the histiocytic population, M_0_ macrophages were prevalent in 9/12 cases, and M_1_ emerged in 2/12; in the remaining cases, we observed a profile corresponding to that of peripheral blood monocytes. The proportions in A indicate the percentage for each cell population in the samples; the p-value was below 0.001 for all the samples.

Since the immunophenotype of LL cases is apparently not consistent with a well-defined T-cell subset (Hartmann et al., 2011), based on the GEP data generated, we used CIBERSORT to quantify the diverse cellular components and assign the neoplastic ones to a specific functional subset. Overall, we found that CD4+ were the most represented family of T-cells; particularly, CD4 naïve profile was the most prevalent in 8/12 samples, CD4 memory activated profile was observed in 2/12 (in one case being the CD8 profile equally abundant), and a T-follicular helper (T_FH_) profile in one sample. In one case, gamma/delta T-cells were the most prevalent T-cells (Figure 1A; **Supplementary Table 1**).

Based on the assumption that most abundant T-cells should correspond to the neoplastic clone, CD4 lymphocytes were likely to be the cellular counterpart of LL. However, due to the heterogeneous population admixing reactive and neoplastic cells, this cannot be definitely stated in all instances.

By looking at the histiocytic population, we found that M_0_ macrophages were prevalent in 9/12 cases, while M_1_ emerged in 3/12 cases (**Figure 1A**). Of note, the observed prevalence patterns were confirmed when we compared the three macrophage phenotypes (**Figure 1B**).

### Lennert Lymphoma Is Similar to but Distinct From Other Peripheral T-Cell Lymphoma—Not Otherwise Specified, Based on the Gene Expression Profiles

**Figure 2:**
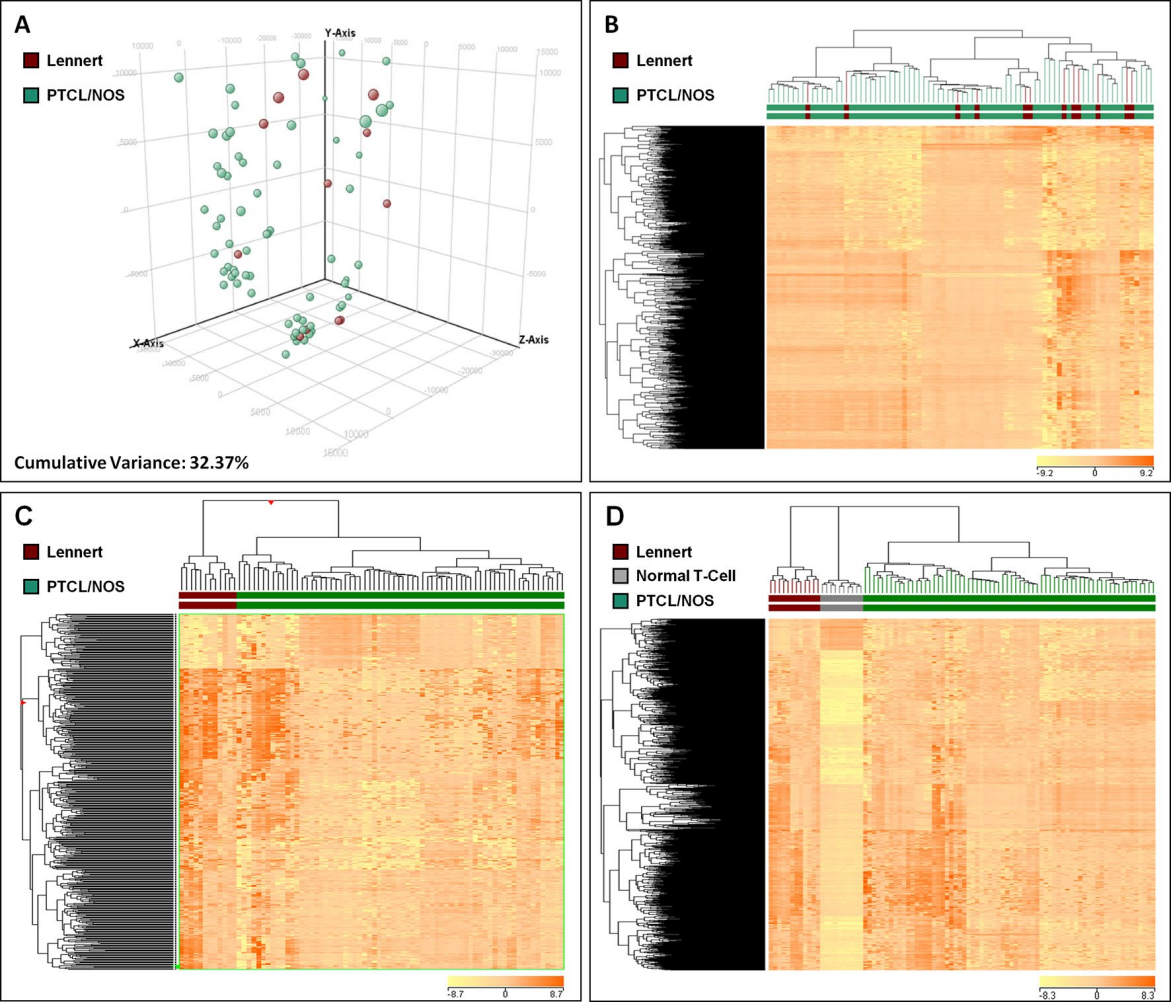
Unsupervised and Supervised analysis on Lennert lymphoma vs. other peripheral T-cell lymphoma—not otherwise specified and normal T-cells. **(A)** Unsupervised principal component analysis showed similar profiles of the two tumor groups. **(B)** HCA indicated no clear distinction of LL and other PTCL/NOS. **(C)** Supervised HCA based on the differentially expressed genes between the two tumor categories resulted in a clear distinction of them. **(D)** Supervised analysis (t-test, FDR-adjusted p ≤ 0.05 and fold change ≥2) identified 622 genes differentially expressed in LL vs. normal T-lymphocytes, most of which were overexpressed in LL. A HCA using the expression levels of those genes resulted in obvious discrimination of the two categories, as well as other PTCL/NOS cases. In **A**, each sphere in the three-dimensional space represents a single sample, and each dimension represents a principal component. In the heat maps **(B, C** and **D)**, each column represents a sample, and each row represents a probeset (gene). The color scale bar shows the relative gene expression changes normalized to the standard deviation (0 is the mean expression level of a given gene).

In order to compare the molecular features of LL with other PTCL/NOS, we analyzed the global GEPs of 12 LL and 68 PTCL/NOS cases. At first, we exploited two widely used unsupervised approaches, namely PCA and HCA. The results of PCA showed a homogeneous dispersion of almost all cases including LLs (**Figure 2A**, **Supplementary Figure 1**), with cumulative variance limited to 32.37%. The results of unsupervised HCA turned out to be very similar to PCA, with no clear distinction of LL among other PTCL/NOS (**Figure 2B**).

To find differentially expressed genes, we then proceeded with direct comparison of LL and other PTCL/NOS. Supervised analysis (T-test, p-value ≤ 0.01, fold change ≥2) identified 455 genes differentially expressed between the two tumor groups, 385 of which were upregulated in LL. These genes clearly discriminated the two settings (**Figure 2C**; **Supplementary Table 2**; **Supplementary Figure 2**).

We then sought to assess whether the genes differentiating LL from other PTCL/NOS cases might have a significant role in tumorigenesis. Interestingly, analysis of the genes upregulated in either LL or PTCL/NOS using Molecular Signatures Database uncovered a significant enrichment in highly relevant processes in LL, such as upregulation of cell differentiation, regulation of apoptosis and immune response; conversely, cell growth/maintenance and cell proliferation turned out to be downregulated in LL, or, otherwise said, upregulated in PTCL/NOS (**Supplementary Table 3**). These processes were speculated to be involved in important terms such as lymphoma development and microenvironment.

### Lennert Lymphoma Differs From Normal T-Lymphocytes Based on Gene Expression Profiles

We then compared LL with normal T-lymphocyte. By supervised analysis, 622 genes were found to be differentially expressed (t-test, FDR-adjusted p-value ≤ 0.005, fold change ≥2), the majority of which were upregulated in LL (**Supplementary Table 4**; **Supplementary Figure 3**). Such signature easily discriminated normal and neoplastic samples at HCA. Interestingly, such signature was able to discriminate PTCL/NOS from LL and normal T-cells as well (**Figure 2D**).

**Figure 3:**
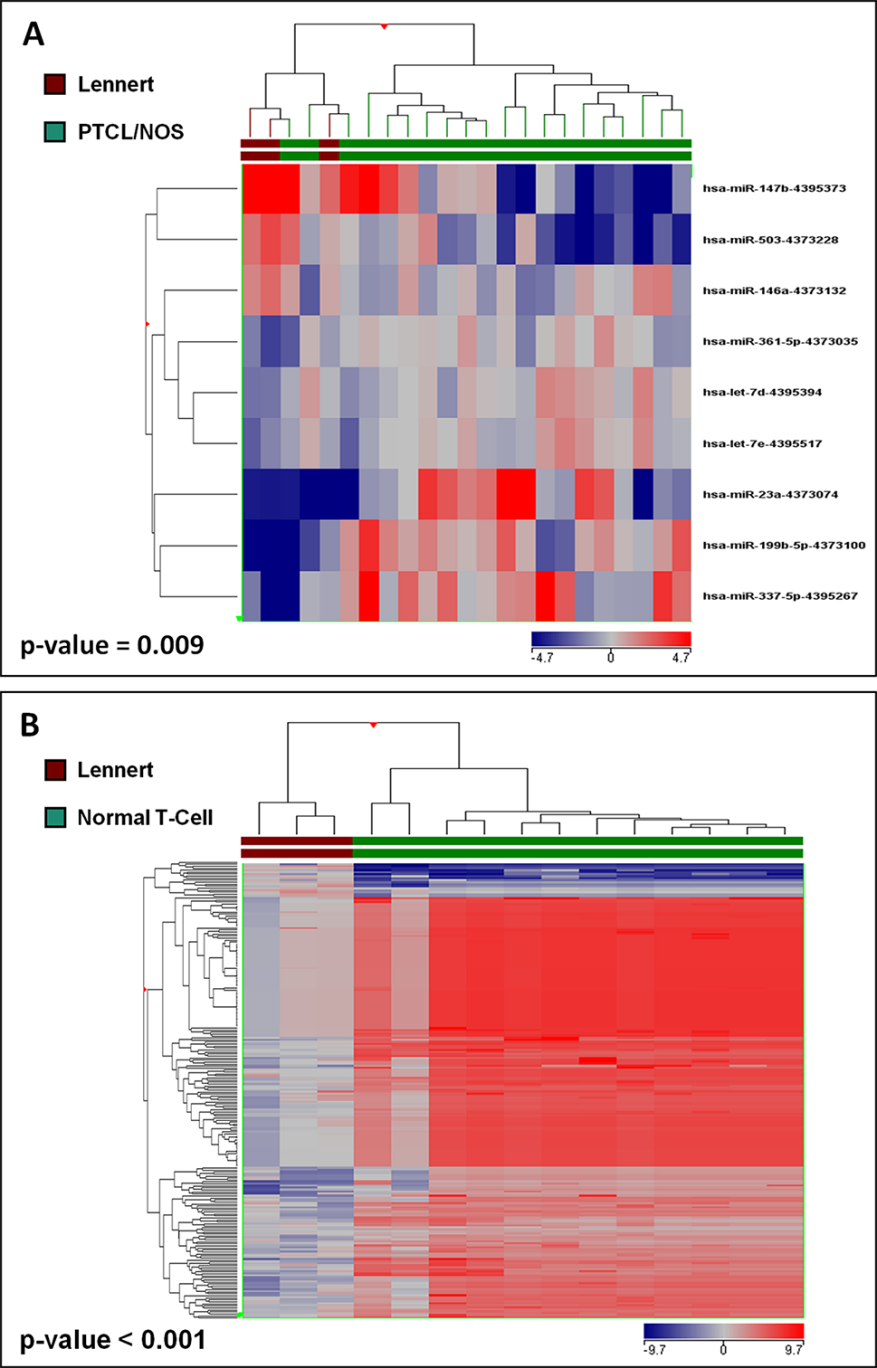
Lennert lymphoma presents a unique microRNA signature. **(A)** Supervised analysis (t-test, p < 0.05 and fold change >2) identified nine miRNA differentially expressed in LL vs. other PTCL/NOS, based on the expression of which, the two diseases were clearly differentiated. **(B)** Supervised analysis (t-test, Benjamini-corrected p < 0.01 and fold change >2) identified 229 miRNA differentially expressed in LL vs. normal T-lymphocytes, and their expression values could discriminate normal and neoplastic samples clearly.

The differentially expressed genes turned out to be significantly enriched in biological processes potentially relevant to malignant phenotype, including cell growth, angiogenesis, cell adhesion/cell–cell signaling; in addition, some programs were likely related to microenvironment, such as response to wounding, collagen catabolism, and extracellular matrix (**Supplementary Figure 3**). Furthermore, direct comparison of the two groups by gene set enrichment analysis also indicated enrichment in Hedgehog signaling, nuclear factor kappa-B and STAT1 targets, phospholipase C activity, and KRAS signaling and response to platelet-derived growth factor in LL (**Supplementary Figure 4**).

**Figure 4:**
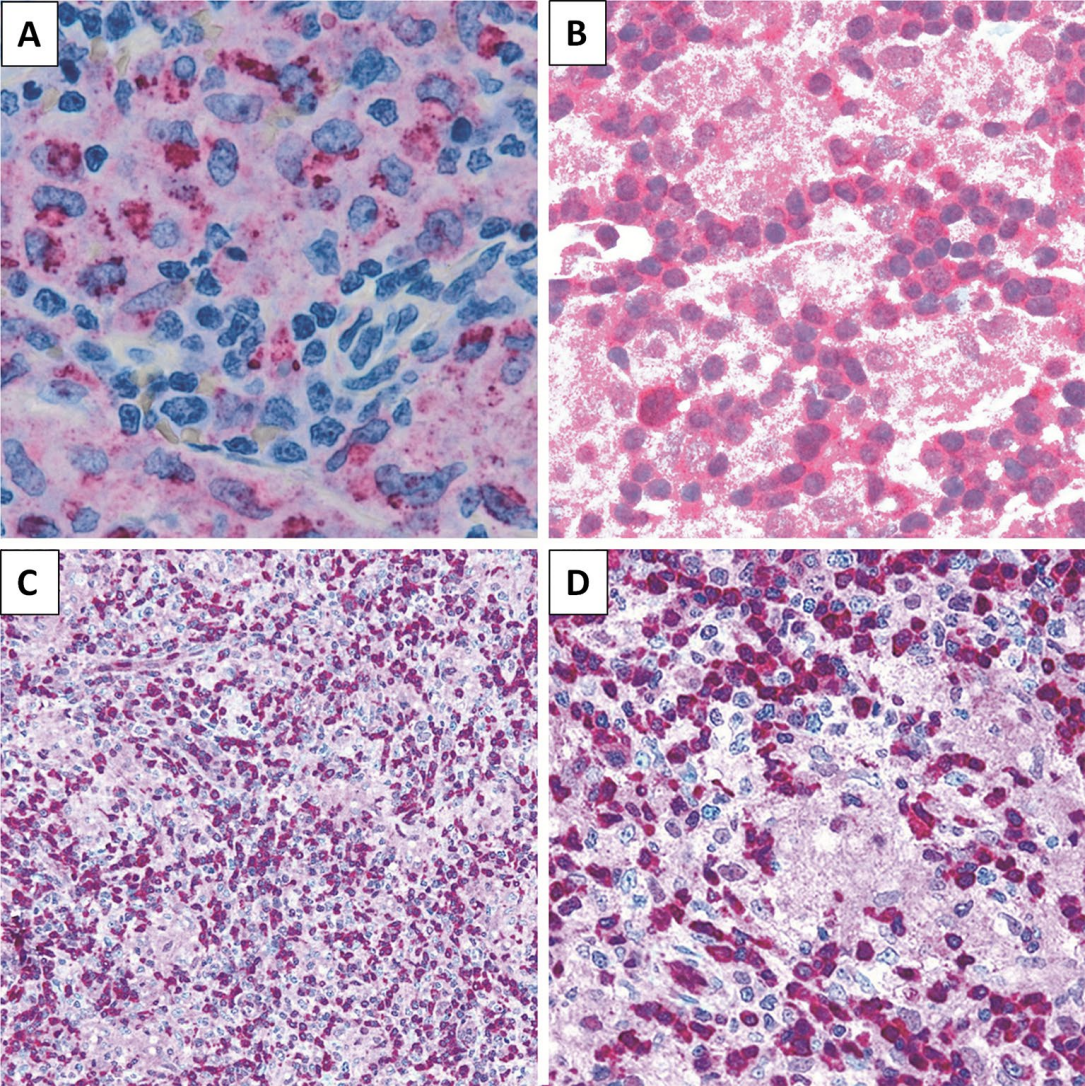
Immunohistochemical staining. LL case showing **(A)** CD68 (PGM-1) expression in epithelioid component (×200), **(B)** STAT5 positivity (×200), and **(C, D)** PDCD4 expression in lymphoid population (**C**, ×200; **D**, ×400).

### MicroRNA and Gene Expression Signatures of Lennert Lymphoma Suggest PI3K/Akt/mTOR Pathway as a Potential Therapeutic Target

To identify deregulated miRNAs in LL, we performed miRNA profiling on 3 LL, 20 PTCL/NOS, and 12 normal CD4+ T-cell cases. By supervised analysis (p-value ≤ 0.05, fold change ≥2, i.e., the most widely used criteria in the literature), we identified nine cellular miRNAs differentially expressed in LL, as compared with other PTCL/NOS (**Table 2**). An HCA based on the expression of the deregulated miRNAs resulted in the two diseases being clearly differentiated (p-value = 0.009, **Figure 3A**). A similar approach was followed, comparing 3 LL and 12 normal T-lymphocyte samples, which identified 229 miRNAs differentially expressed (t-test, FDR-adjusted p-value < 0.01, fold change >2), most of which were downregulated in LL (**Supplementary Table 5**), and a clear classification of the normal and malignant samples was obtained in a HCA (**Figure 3B**).

**Table 2:** MicroRNAs differentially expressed in Lennert lymphoma vs. peripheral T-cell lymphoma—not otherwise specified.

**Target Id**	**p-value**	**Fold Change**	**Regulation in Lennert Lymphoma**
hsa-let-7d-5p	0.012553142	2.7276673	down
hsa-let-7e-5p	0.011690697	3.0097759	down
hsa-miR-23a-3p	0.027455721	28.169153	down
hsa-miR-146a-5p	0.019856786	3.381771	up
hsa-miR-147b	0.022377128	37.29126	up
hsa-miR-199b-5p	0.005994446	20.567883	down
hsa-miR-337-5p	0.017264001	16.229063	down
hsa-miR-361-5p	0.006049584	3.3813345	down
hsa-miR-503-5p	0.022841154	14.025469	up

To better understand the possible pathogenic potential of deregulated miRNAs between LL and other PTCL/NOS, we identified their target genes that were already biochemically validated (**Supplementary Table 6**). Among them, however, only four were differentially expressed in LL and PTCL/NOS, while a higher number of them (n = 7) could be related to genes differentiating LL and normal T-lymphocytes (**Supplementary Figure 5**).

Interestingly, according to pathway analysis, performed by IPA software based on the combination of differentially expressed miRNA and genes, PI3K/Akt/mTOR signaling axis, a well-described pathway in human malignancies, was suggested to have a central role in molecular pathology of LL when compared with normal T-cells, acting *via* STAT5 (**Supplementary Figure 6**).

### PDCD4 and STAT5 Are Expressed in Lennert Lymphoma

We then sought to assess the expression of selected proteins emerged from GEP analysis, aiming to confirm the obtained results. In particular, by means of IHC, we studied PDCD4 and STAT5 for their potential expression in the neoplastic clone as well as CD68 as representative of microenvironmental molecules. We found LL to express PDCD4 and STAT5 at protein level. As expected, the epithelioid component resulted CD68 (PGM-1)-positive (**Figure 4**).

## Discussion

LL, the most common morphological variant of PTCL/NOS, displays morphological characteristics, such as prevalent proliferation of small lymphoid cells and dense clusters of epithelioid histocytes, and cytotoxic T-cell phenotype of the majority of cases (Swerdlow et al., 2016). Being a rare disease, there is scarce amount of information on the biological nature of LL (Quintanilla-Martinez et al., 1994). Currently, LL is diagnosed only based on morphology and immunohistochemical characteristics. Some researchers, however, have suggested that LL might actually be a distinct entity, and few evidences support this hypothesis including the particular morphology and the relatively favorable clinical course (Weisenburger et al., 2011). Recently, in the last edition of the WHO classification, PTCL/NOS with follicular morphology, previously recorded as PTCL/NOS morphological variants, was regarded as a separate category (the so-called follicular T-cell lymphoma) and based on phenotypic and molecular evidences grouped with other T-follicular helper-related lymphomas (Swerdlow et al., 2016).

Here, for the first time, we present evidences obtained from studying the molecular profile of LL concerning both gene and miRNA expression signatures that support the classification of LL as a separate disease. The cellular counterpart of LL, one criteria that is currently most considered for tumor classification, has been largely debated being still unsolved. There were some weak evidences that suggest that the major part of LL could be originated from cytotoxic T-cells. For example, Geissinger et al. performed IHC on 101 PTCL/NOS cases that include 18 LLs and represented LL is mainly derived from CD8+ cytotoxic cells (Geissinger et al., 2004). Furthermore, Hartmann et al. studied on 97 epithelioid cell-rich lymphomas that include 17 LLs, and they showed that the most LL cases extremely express cytotoxic T-cell markers and are different from other epithelioid cell-rich lymphomas, although CD8 was not consistently expressed with most tumors being CD4+ (Hartmann et al., 2011). Consistent with the latter, a recent study indicated LL to correspond to CD4+ cells based on the immunophenotype, though cytotoxic markers can be expressed (Kurita et al., 2016). Of note, our analysis by CIBERSORT indicated that the most prevalent population, beside the histiocytic components, was represented by CD4+ lymphocytes, while CD8 and gamma–delta ones were indeed limited. Although this does not represent a formal demonstration of correspondence between LL and CD4+ cells, in combination with the most recent immunophenotypic analysis, it certainly and strongly supports such hypothesis.

We compared the global gene signatures of all PTCL/NOS, trying to define the general position of LL. The unsupervised methods failed a clear discrimination of LL and other PTCL/NOS, indicating a global similarity among them. However, a more stringent comparison of LL and other PTCL/NOS, accomplished using supervised methods, supported the existence of different molecular signatures at some level. The differentially expressed genes turned out to be engaged in malignancy-related processes, probably pointing at some different tumorigenesis mechanisms in the two groups. Consistent with the less aggressive clinical course, we found that tumor proliferation was reduced when compared with other PTCL/NOS. Rather, deregulation of apoptosis was indicated. Furthermore, enrichment in cell to cell communication may suggest that the abundant microenvironmental components may have a role in supporting tumor growth (Brucher and Jamall, 2014). The gained signature could potentially classify the two categories correctly. However, in order to prove the validity of such signature, further independent studies are needed.

Inspired by the results obtained so far, and considering the important role of miRNAs in lymphomagenesis (Navari et al., 2018), we decided to expand our analysis to the miRNA signature of LL as compared with other PTCL/NOS. Although having access to a limited number of LL cases for this aim, we could find some miRNAs differentiating the two, but again the number was limited, which once again made the similar nature of the two diseases standing out. The resulted miRNAs, furthermore, were capable of separating the two set of samples in an HCA. Of note, we have previously reported the usage of miRNA profiling in classifying PTCL subtypes (Laginestra et al., 2014).

Next, we compared miRNA signature of LL with normal T-cells, with the intention of discovering the underlying pathological mechanism of LL. Compared with what we observed in the case of LL vs. other PTCL/NOS, here, we found a much higher number of differentially regulated miRNAs, which could be a result of contrasting tumor vs. normal cells, as compared with contrasting tumor vs. tumor in the previous case. Importantly, few of the miRNAs seemed to be overexpressed in LL, which corresponds to the previous reports regarding a globally lowered expression of human miRNAs in the tumors (Suzuki et al., 2012; Peng and Croce, 2016; Ramassone et al., 2018). Our previous experiences indicated that the direct comparison of FFPE and fresh samples using DASL array can be accomplished with reliable results (Sapienza et al., 2014), thus a similar approach was carried out on the GEPs of LL and normal T-cells. Very interestingly, we found the major part of deregulated genes to have increased expression level in LL. This would be consistent with the linkage between gene and miRNA expression, and this would suggest at least some of those genes to be direct or indirect targets of the downregulated miRNAs in LL. Again, the number of the differentially expressed genes was higher when compared with LL vs. other PTCL/NOS, and the deregulated genes, as anticipated, were involved in relevant tumorigenesis processes, most notably microenvironment as well as neoplastic clone. Analysis of CD68 expression using IHC confirmed the validity of our results and promoted the inflammation induced by tumor-associated macrophage as an underlying tumorigenesis mechanism in this cancer (Liu et al., 2014).

Furthermore, we looked at the expression of two important molecules related to cancer. STAT5 is a member of the STAT family of transcription factors, the activation of which has been shown to be essential in peripheral T-cell lymphomas, promoting it as a possible therapeutic target (Simpson et al., 2018), Although PDCD4 is generally considered as a tumor suppressor, however, pro-inflammatory roles have been suggested for it as well (Young et al., 2010). We found both of these genes to be expressed in LL at protein level. Very interestingly, we identified a downregulation of hsa-miR-23a-3p in LL, which is reported to directly target PDCD4 (Hu et al., 2017). More research is waranted in order to shed more light upon the role of these genes and especially PDCD4 in LL and other PTCLs.

Since our results of both miRNA and GEPs of LL seemed to be correlated with its pathogenesis, we used them to create a network of correlated cell signaling pathways using IPA, which resulted in the discovery of PI3K/Akt/mTOR axis as the most relevant pathway. Although using an *in silico* approach, Martin-Sanchez et al. recognized this pathway to be activated in PTCLs, this is for the first time that this hypothesis is suggested for LL (Martín-Sánchez et al., 2013). We tried to test this idea experimentally; however, the lack of a cell line derived from LL turned out to be a major obstacle. Treatment of PTCL patients with the inhibitors of PI3K/Akt/mTOR axis has been suggested by others, but, again, no data focused on LL are available (Martín-Sánchez et al., 2013; Blachly and Baiocchi, 2014; Islam et al., 2017; Horwitz et al., 2018). It is worth to mention that the role of tumor-associated macrophages in promoting PI3K/Akt/mTOR signaling in malignant cells has been reported (Dwyer et al., 2017; Zhang et al., 2017; Zheng et al., 2018; Ye et al., 2018), which corresponds to our results regarding the role of this signaling in LL. Since no LL cell lines are available, formal experimental support could not be provided; however, the potential role of PI3K/Akt/mTOR inhibitors is under investigation in other PTCL subtypes (Martín-Sánchez et al., 2013; Horwitz et al., 2018).

Since by far we found molecular evidences supporting LL as a distinct entity, we tried to analyze the clinical data, as a complementary approach. We report, for the first time, a significant difference of male: female ratio and B-symptoms in LL, as compared with other PTCL/NOS. However, although a trend of better treatment response and survival rate was found for LL in comparison with other PTCL/NOS, our results were not statistically significant. Nonetheless, this might be due to the limited number of the patients analyzed, since a large international study reported a better overall survival in LL as compared with other PTCL/NOS (Weisenburger et al., 2011).

In conclusion, we provided genomic data supporting classification of LL as a distinct entity, rather than simply a morphological variant of PTCL/NOS. Although a distinct molecular profile per se is not sufficient for defining a pathological entity, our data are complementary to previous phenotypic and clinical analyses (Hartmann et al., 2011; Weisenburger et al., 2011). Indeed, the correct recognition of this PTCL subtype may be relevant in the future for specific targeted therapy and warrants further clinical evaluation in an international contest. Furthermore, development of model cell lines and animals for LL and other PTCL/NOS along with genome-wide analysis of a broader number of cases would improve our understanding of this challenging group of malignancies.

## Data Availability

The data has been uploaded in our institutional repository, and is available to the editors and referees. The data would be publicly available upon the publication of this manuscript along with other two related manuscripts.

## Author Contributions

All the authors collected and analyzed the data. ME, MN, and PP wrote the manuscript. PP was responsible for the conception and design of the study.

## Funding

This work was supported by BolognAIL (Prof. Piccaluga), RFO (2014 and 2018, PP), AIRC (PP IG 2013 N.14355), and FIRB (Futura 2011 RBFR12D1CB, PP).

## Conflict of Interest Statement

The authors declare that the research was conducted in the absence of any commercial or financial relationships that could be construed as a potential conflict of interest.
