# Supplementary material for: Transcriptional Analysis of Lennert Lymphoma Reveals a Unique Profile and Identifies Novel Therapeutic Targets
Source: Front Genet. 2019 Sep 10;10:780. doi: 10.3389/fgene.2019.00780 (PMC6748072; doi:10.3389/fgene.2019.00780)
Supplement: Supplementary file 7 [file Presentation_1.pdf]

Supplementary Material

1 Supplementary Figures

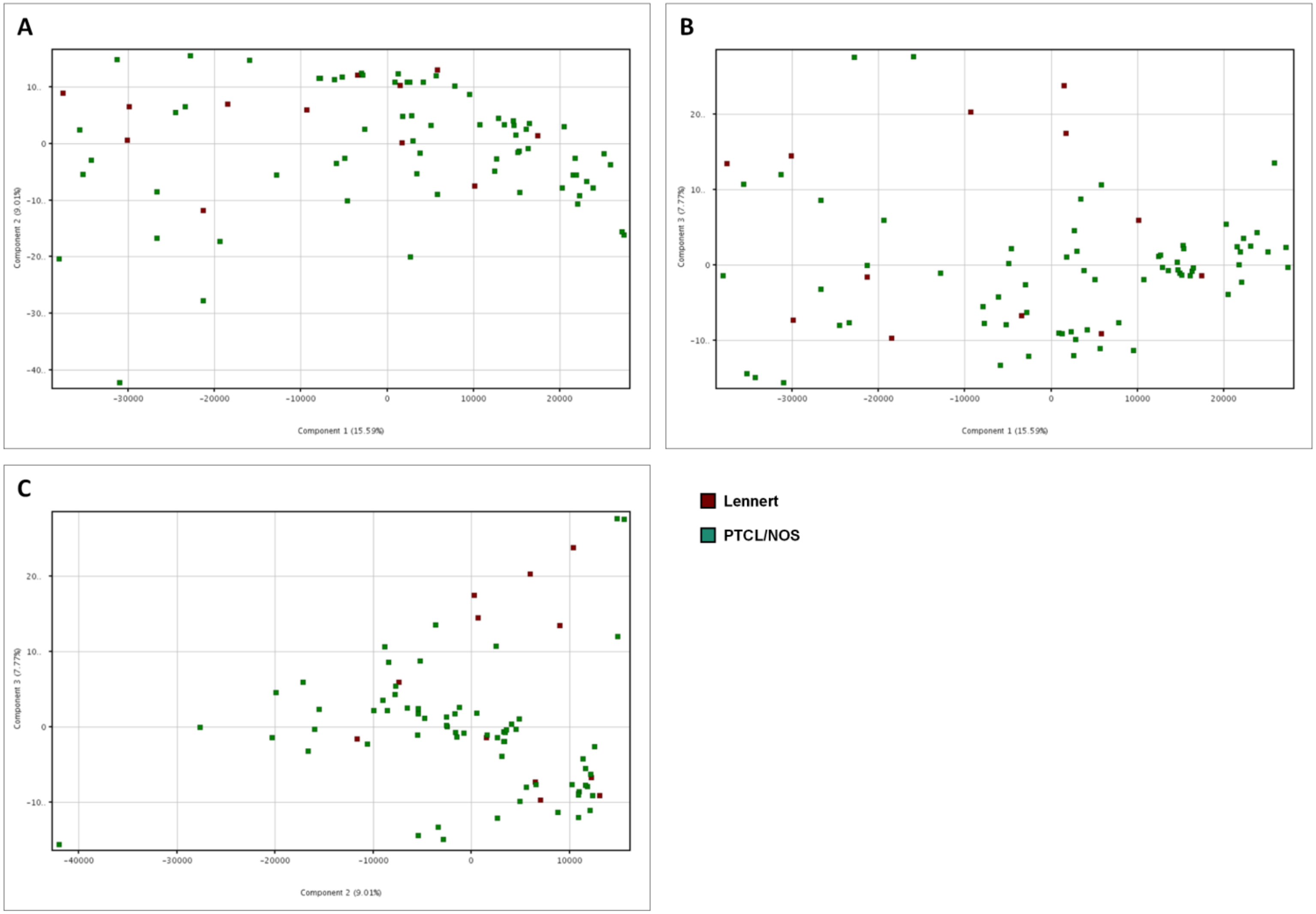

Supplementary Figure 1. Two dimensional representation of principal component analysis (PCA) performed on GEP of Lennert Lymphoma and PTCL/NOS cases. This analysis (A: principal component 1 vs. principal component 2, B: principal component 1 vs. principal component 3, C: principal component 2 vs. principal component 3) indicated similar profiles for the two tumor categories.

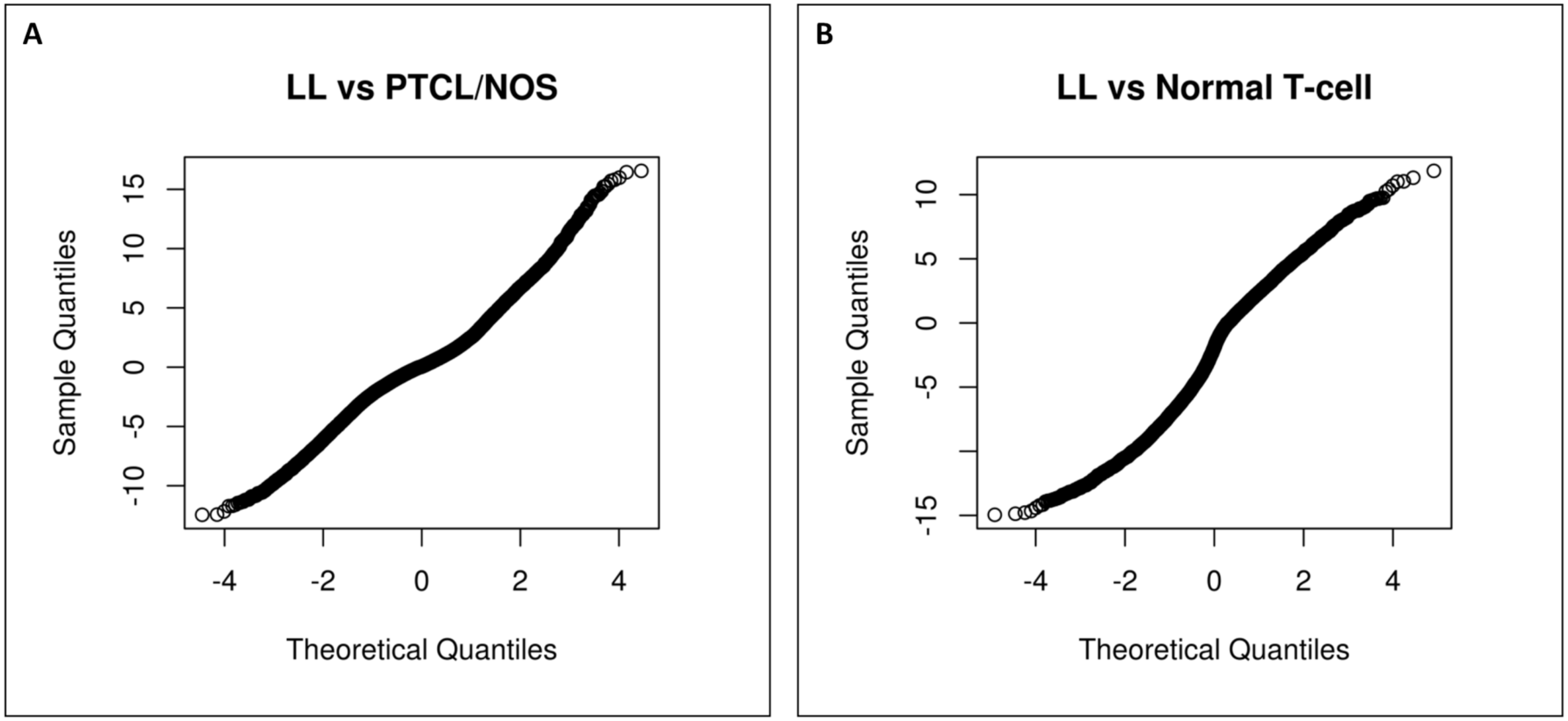

Supplementary Figure 2. Q-Q plot for the genes differentially expressed. The plot was generated for genes differentially expressed between LL vs. other PTCL/NOS **(A)** and between LL and normal T-cells **(B)**. The plots were produced and depicted based on the original data, i.e. log2 transformed.

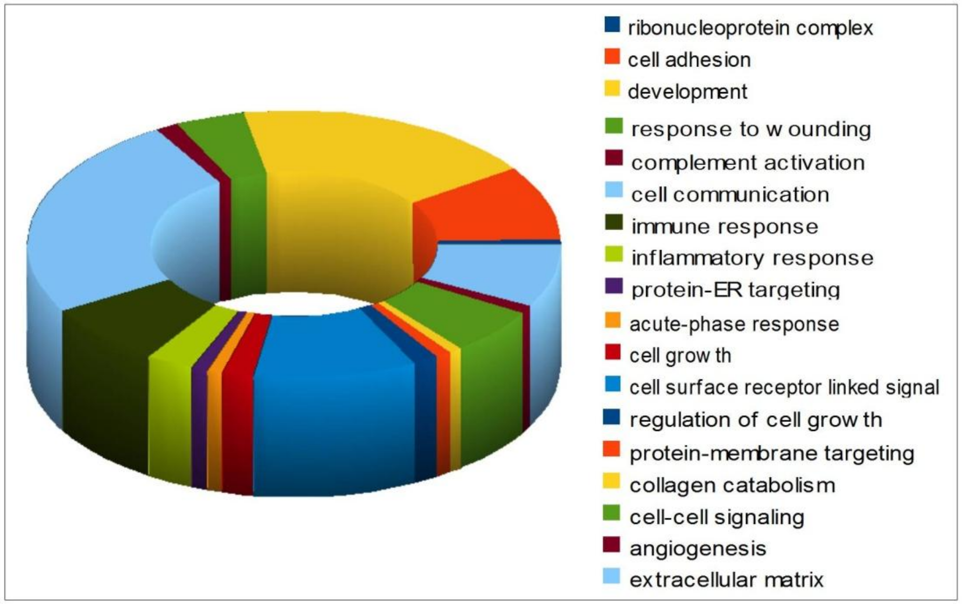

Neoplastic clone
ribonucleoprotein complex
cell growth
cell surface receptor linked signal transduction
protein-membrane targeting
angiogenesis
cell adhesion/cell-cell signaling

Microenvironment
response to wounding
acute-phase response
collagen catabolism
extracellular matrix

Undefined
Immune response

Supplementary Figure 3. Gene Onthology enrichment analysis by MSigDB tool based on the genes differentially expressed between Lennert Lymphoma and normal T-cells. This analysis revealed a significant enrichment in relevant pathways and cellular programs that could reasonably be referred to either the neoplastic clone or to the microenvironment or to both. The proportions indicate the number of the genes in each category overrepresented in our dataset. The p-value for all the terms is below 0.05.

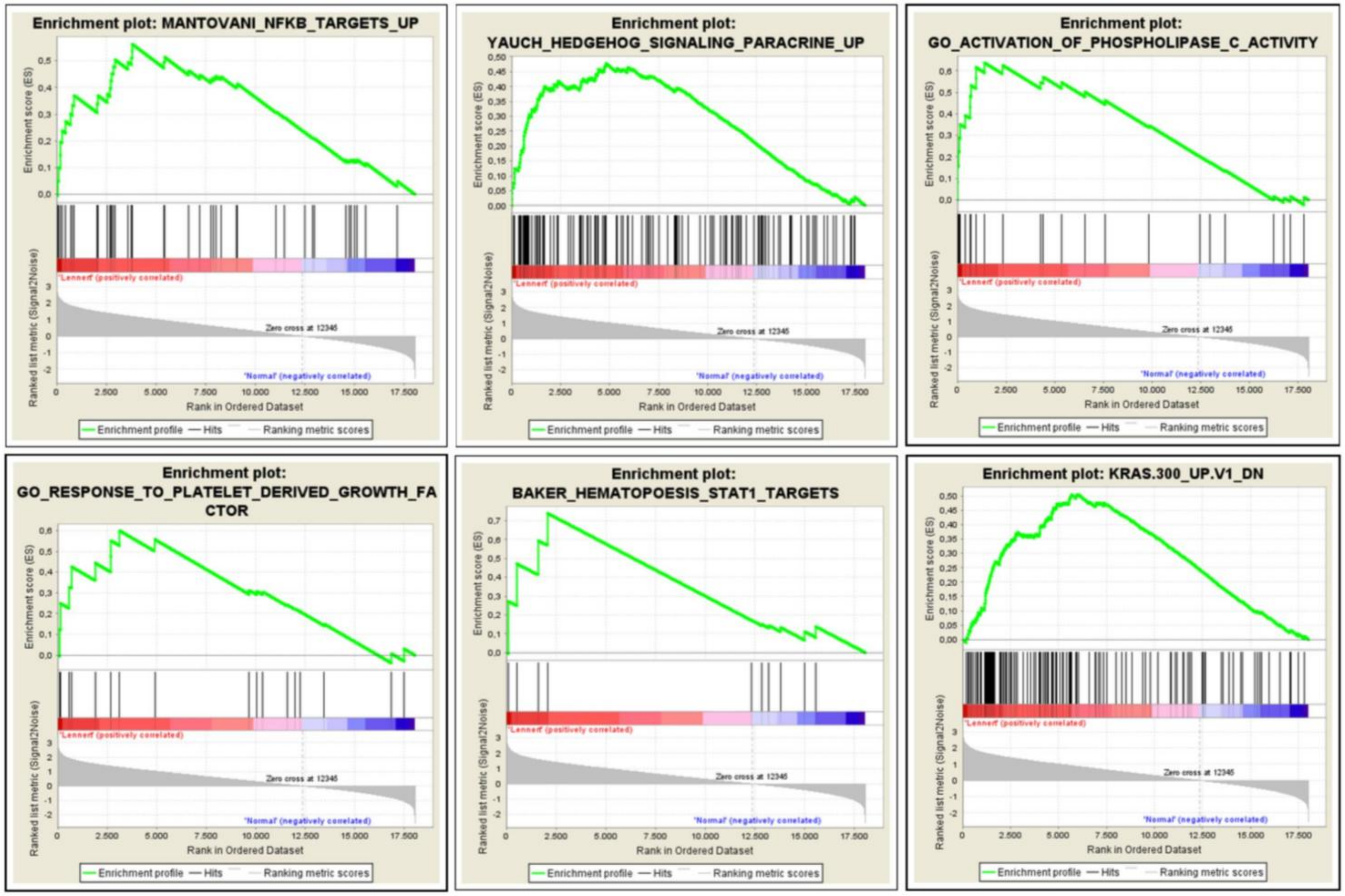

Supplementary Figure 4. Direct comparison of Lennert lymphoma (LL) with normal T-lymphocytes by means of GSEA. We found enrichment in Hedgehog signaling, nucleo factor kappa-B (NF-kB) and STAT1 targets, phospholipase C activity, KRAS signaling, and response to platelet derived growth factor in LL.

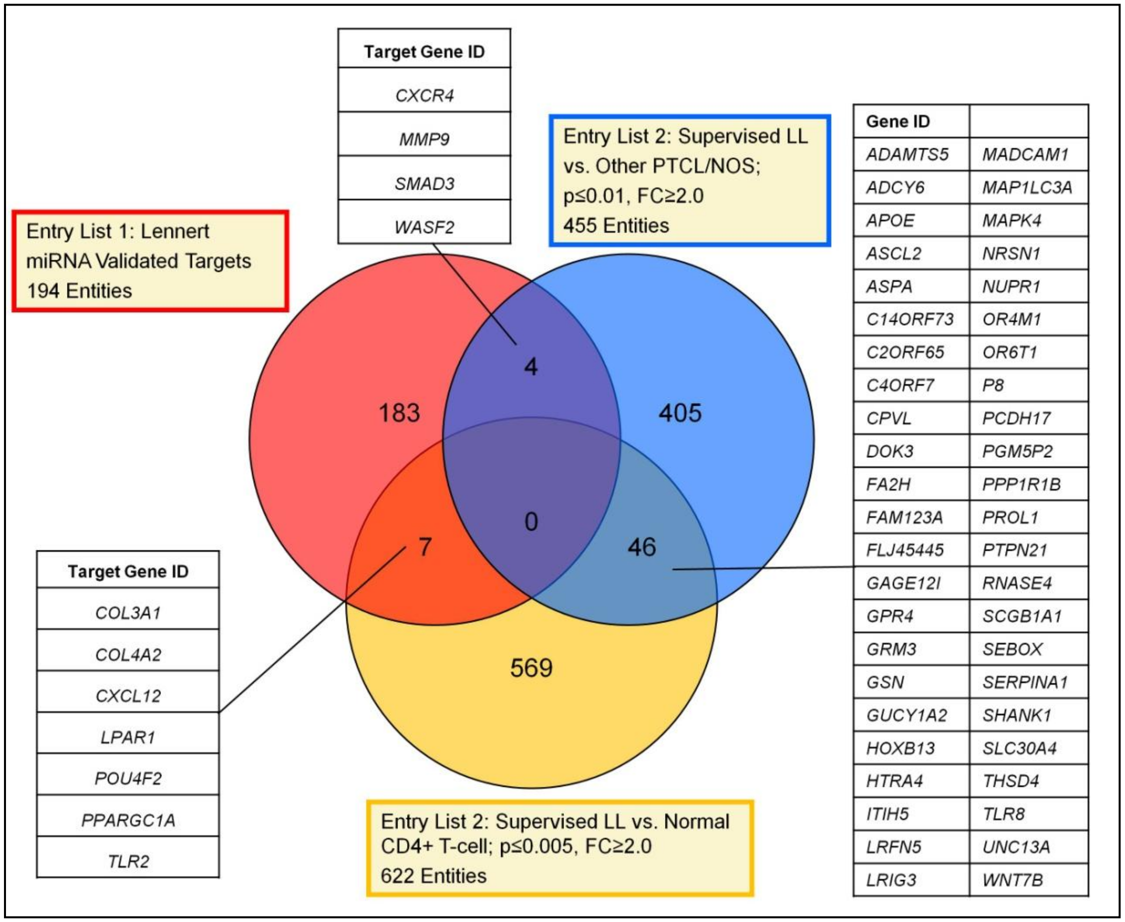

Supplementary Figure 5. miRNAs target genes related to genes differentiating Lennert Lymphoma (LL) vs. other PTCL/NOS and normal T-lymphocytes. The figure represents the overlap (Venn diagram) between validated targets of miRNAs deregulated in LL, differentially expressed between LL vs. other peripheral T-cell lymphoma (PTCL/NOS) and differentially expressed between LL and normal CD4+ T-cell.

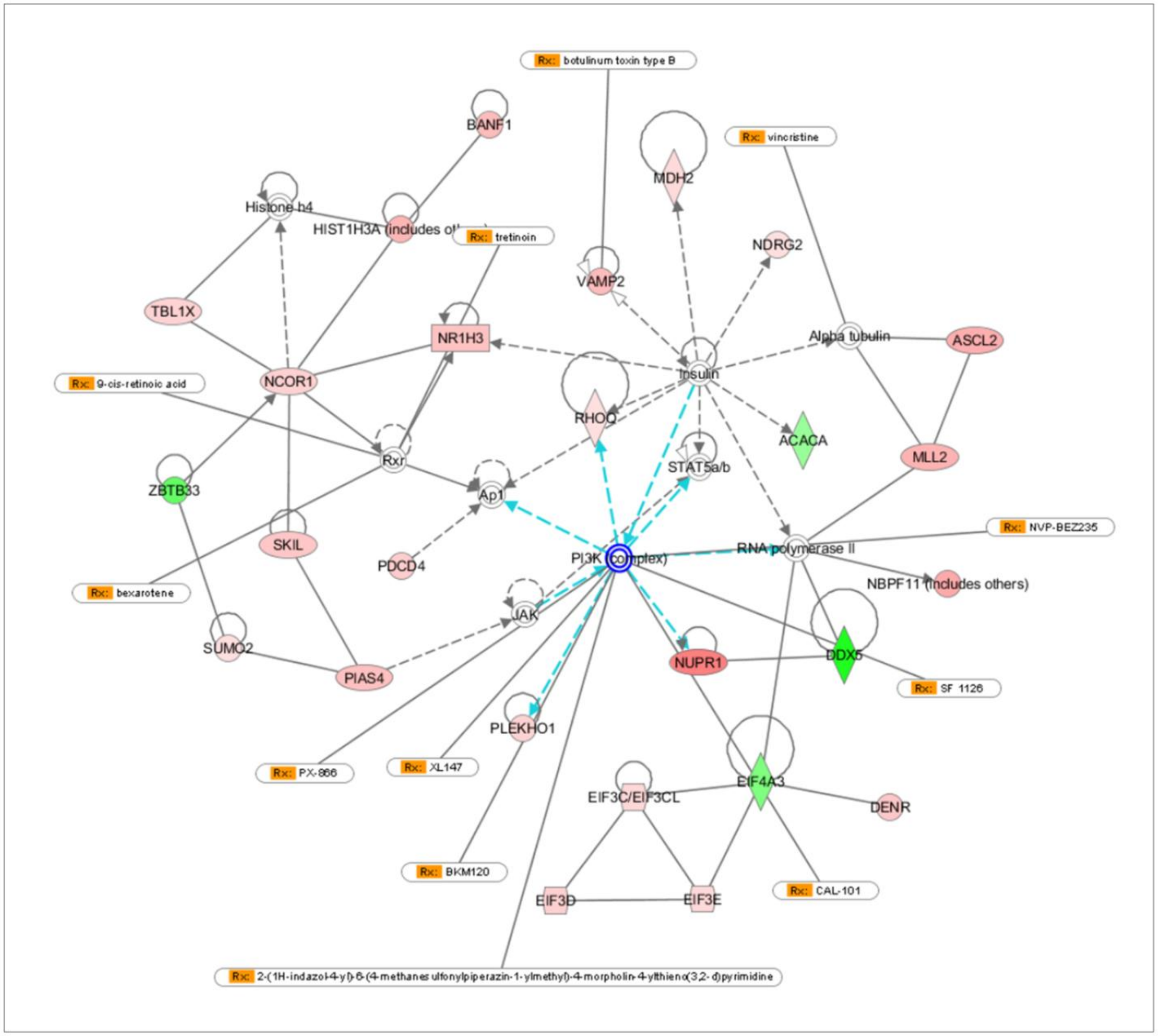

Supplementary Figure 6. The MTOR/PI3K pathway is a suitable therapeutic target in LL. By means of Ingenuity Pathway Analysis (IPA) performed on genes and miRNA deregulated in Lennert lymphoma, the PI3K/STAT5 axis was identified as possible therapeutic target.
